# A microRNA profile of human CD8^+^ regulatory T cells and characterization of the effects of microRNAs on Treg cell-associated genes

**DOI:** 10.1186/s12967-014-0218-x

**Published:** 2014-08-06

**Authors:** Fadi Jebbawi, Hussein Fayyad-Kazan, Makram Merimi, Philippe Lewalle, Jean-Christophe Verougstraete, Oberdan Leo, Pedro Romero, Arsene Burny, Bassam Badran, Philippe Martiat, Redouane Rouas

**Affiliations:** Experimental Hematology, Institut Jules Bordet, Université Libre de Bruxelles, 121, Boulevard de Waterloo, 1000 Bruxelles, Belgium; Department of Gynecology and Obstetrics, Clinique Saint-Pierre, Ottignies, Belgium; Ludwig Center for Cancer Research of the University of Lausanne, Lausanne, Switzerland; Department of Biochemistry, Laboratory of Immunology, EDST-PRASE, Faculty of Sciences, Lebanese University, Hadath-Beirut, Lebanon

**Keywords:** Regulatory T cells, microRNA, FOXP3, CTLA-4, GARP, CD8

## Abstract

**Background:**

Recently, regulatory T (Treg) cells have gained interest in the fields of immunopathology, transplantation and oncoimmunology. Here, we investigated the microRNA expression profile of human natural CD8^+^CD25^+^ Treg cells and the impact of microRNAs on molecules associated with immune regulation.

**Methods:**

We purified human natural CD8^+^ Treg cells and assessed the expression of FOXP3 and CTLA-4 by flow cytometry. We have also tested the ex vivo suppressive capacity of these cells in mixed leukocyte reactions. Using TaqMan low-density arrays and microRNA qPCR for validation, we could identify a microRNA ‘signature’ for CD8^+^CD25^+^FOXP3^+^CTLA-4^+^ natural Treg cells. We used the ‘TargetScan’ and ‘miRBase’ bioinformatics programs to identify potential target sites for these microRNAs in the 3′-UTR of important Treg cell-associated genes.

**Results:**

The human CD8^+^CD25^+^ natural Treg cell microRNA signature includes 10 differentially expressed microRNAs. We demonstrated an impact of this signature on Treg cell biology by showing specific regulation of FOXP3, CTLA-4 and GARP gene expression by microRNA using site-directed mutagenesis and a dual-luciferase reporter assay. Furthermore, we used microRNA transduction experiments to demonstrate that these microRNAs impacted their target genes in human primary Treg cells ex vivo.

**Conclusions:**

We are examining the biological relevance of this ‘signature’ by studying its impact on other important Treg cell-associated genes. These efforts could result in a better understanding of the regulation of Treg cell function and might reveal new targets for immunotherapy in immune disorders and cancer.

## Background

Regulatory T (Treg) cells are specialized subsets of T cells that modulate the immune system to avert unwanted immune responses, maintain immunological self-tolerance and homeostasis, dampen inflammatory responses, and limit tissue damage. Treg cells can be divided into natural and adaptive Treg cells. Natural Treg cells develop in the thymus and express both CD25 and FOXP3. Adaptive Treg cells develop in the periphery following antigenic (by self- or foreign-antigen) stimulation in the presence of specific immunomodulatory molecules. Moreover, Treg cells can also be divided into two classes, CD4^+^ and CD8^+^ Treg cells [1].

CD8^+^ T cells play a major role as adaptive effectors in several immunopathological conditions, such as autoimmune disease [2-6], transplantation [7,8], host defense and cancer [8,9]. Despite the long-held notion that CD8^+^ T cells could mediate suppression, most reports have focused on the regulatory properties of CD4^+^ T cell subsets and only a few studies have characterized CD8^+^ T cell-mediated immune regulation. The main reason for the relative neglect of CD8^+^ Treg cells by researchers is the absence of markers that allow their identification. Recently, several subsets of CD8^+^ Treg cells have been identified based on the expression of CD25, CD56 [10], FOXP3, CXCR3, LAG-3, CD103, CD122 and/or HLA-G, as well as the absence of CD28 expression. These diverse subpopulations of CD8^+^ Treg cells have different mechanisms of suppression and play roles in cancer, infection, transplantation and autoimmunity [11-25]. Natural CD8^+^ Treg cells include CD8^+^CD25^+^ T cells, which share functional and phenotypic similarities with CD4^+^CD25^+^ Treg cells, such as CTLA-4, GITR and intracellular Foxp3 expression. CD8^+^CD25^+^ Treg cells can suppress CD4^+^CD25^−^ T cells in a membrane-bound TGFβ and CTLA-4-mediated contact-dependent manner that induces IL-2Rα downregulation on target T cells [19]. They can also act by producing immunosuppressive cytokines, such as TGF-β and IL-10 [26], or by inactivating dendritic cells [21]. Moreover, naturally occurring human CD8^+^CXCR3^+^ T cells have been shown to secrete IL-10 and suppress IFN-γ production, in a manner similar to murine CD8^+^CD122^+^ Treg cells [27].

FOXP3 has emerged as a marker for T cells with regulatory activity. Deletion or mutation of FOXP3 is associated with the lymphoproliferative disorder that occurs in *scurfy* mice and immunodysregulation, polyendocrinopathy, enteropathy, X-linked (IPEX) syndrome in humans [28-30]. As a ‘master transcription factor’, FOXP3 is a critical regulator of CD4^+^CD25^+^ Treg cell development and function, and appears to be the best marker to identify natural CD4^+^ Treg cells [31,32]. However, despite being the most specific marker of Treg cells, together with elevated expression of the high-affinity IL-2 receptor-α chain (CD25), FOXP3 cannot be used to isolate viable Treg cells because of its intracellular expression.

Although we still lack specific markers, many cell-surface molecules have been reported to characterize human Treg cells, such as expression of glucocorticoid-induced tumor necrosis factor receptor (GITR), CD62 ligand (CD62L), OX40 (CD134), cytotoxic T-lymphocyte antigen-4 (CTLA-4), and low expression of IL-7 receptor (CD127) [33-35] and glycoprotein A repetitions predominant (GARP) [36]. CTLA-4 is known to be a critical regulator of immune responses by reducing T cell activation and proliferation. CD4^+^ Treg cells are known to constitutively express CTLA-4 [33]. Polymorphisms in CTLA-4 have been associated with several autoimmune diseases, including systemic lupus erythematosus and insulin-dependent diabetes mellitus; a general susceptibility to autoimmune diseases has also been described for CTLA-4 polymorphisms [37-39], emphasizing its pivotal role in immune tolerance.

GARP appears to be a crucial membrane-anchored receptor for latent TGF-β on the Treg cell surface [40,41]. GARP expression has been shown to identify selectively activated human FOXP3^+^ Treg cells and to play a role in Treg cell-mediated immunosuppression [36].

The microRNAs (miRNAs) are an abundant class of evolutionarily conserved small non-coding RNAs that regulate gene expression post-transcriptionally by affecting the degradation and translation of target mRNA transcripts. The biogenesis of miRNAs involves several processing steps that have mostly been defined in cell-based and biochemical studies. Primary miRNA transcripts are first processed into precursor microRNA (pre-miRNA) by the nuclear RNase III enzyme Drosha [42-45]. These pre-miRNAs are then actively transported by Exportin-5 to the cytoplasm, where they are further processed by the cytoplasmic RNase III enzyme Dicer [46-48]. The functional miRNA strand is then selectively loaded into the RNA-induced silencing complex (RISC) [49,50]. Mature miRNAs then guide the RISC to cognate target genes, and target gene expression is repressed by either destabilizing the target mRNAs or repressing their translation. To date, a rapidly growing number of miRNAs have been identified in mammalian cells and shown to be involved in a range of physiological responses, including development, differentiation and homeostasis [51-53].

Recent publications have provided compelling evidence that miRNAs are highly expressed in Treg cells, and that the expression of Foxp3 is controlled by miRNAs. Among miRNAs, miR-21, −24, −31, −95, −210 [51] and −155 [54] affect Foxp3 expression, and miR-155 is an important regulator of lymphocyte function and homeostasis. Other studies have shown that miRNAs are involved in the regulation of T cell function. For example, miR-142-3p can regulate GARP expression in CD4^+^CD25^+^ T cells [55]. Huang et al. showed an indirect effect of miR-142-3p on FOXP3 expression by targeting AC9 mRNA [56]. Moreover, miR-17-92 has been implicated in the regulation of IL-10 secretion by regulatory T cells [57]. Many studies have reported links between alterations in miRNA homeostasis and pathological conditions, such as cancer, cardiovascular disease, diabetes, psychiatric disorders and neurological diseases [58].

Here, we investigated the miRNA expression profile of human natural CD8^+^CD25^+^ Treg cells and its potential impact on Treg cell-associated functional molecules. We focused on a subset of CD8^+^ Treg cells in human cord blood, which contains a distinct population of CD8^+^CD25^+^ Treg cells that are less heterogeneous than in adult peripheral blood. Cord blood is useful for studies aimed at understanding human natural Treg cells because, in contrast to adult blood, they are less contaminated by activated T cells that express CD25 and lack regulatory function [59]. Therefore, we investigated the miRNA expression profile of these natural CD8^+^CD25^+^ Treg cells and compared it with that of CD8^+^CD25^−^ T cells. Additionally, we focused our study on genes that have been reported in the literature to be associated with human Treg cell biology. Interestingly, we found that some miRNAs have direct effects on FOXP3 and CTLA-4 expression, molecules that regulate Treg cell development and function, and also on GARP expression.

## Materials and methods

### Collection and preparation of cord blood samples

After approval by local and academic ethic committees and informed consent, umbilical cord blood mononuclear cells (UCBMC) were isolated from the umbilical vein blood from normal full-term deliveries placenta. UCBMC were isolated by appropriate centrifugation over a lymphocyte separation medium (PAA laboratories). The study was approved by the Institut Jules Bordet scientific, and ethic committees and by the committees of Université Libre de Bruxelles.

### Isolation of T-cell populations

Cord blood CD8^+^ lymphocytes were purified using CD8^+^ T cell isolation kit (Miltenyi Biotec, Bergisch Gladbach, Germany) according to the manufacturer protocol. Briefly, UCBMC were first incubated in PBS supplemented with 2% heat-inactivated fetal bovine serum and saturating amounts of biotin-conjugated antibody cocktail (anti- CD4, CD15, CD16, CD19, CD36, CD56, CD123, TCR-γ/δ and anti-Glycophorin A). Leucocytes were then incubated with anti-biotin microbeads and CD8^+^ T cells were purified using magnetic separation columns (Miltenyi Biotec). The negatively selected cells were incubated with anti-CD25 micro-beads, and then CD25^+^ and CD25^−^ cells were purified from CD8^+^ T lymphocyte fraction using magnetic columns (Miltenyi Biotec).

### FOXP3 intracellular staining and flow cytometry

FOXP3 intracellular staining was performed using anti-human FOXP3-PE detection Kit (BD Biosciences) following the manufacturer's instructions. Anti-CD3-PerCP, anti-CD8-APC, anti-CTLA-4-PE (BD Biosciences), anti-CD25-PE (Miltenyi Biotec) were used to assess cell phenotype and purity. Corresponding isotype controls served as controls. Flow cytometry analysis was performed on a FACSCalibur machine with Cell Quest software (Becton-Dickinson Biosciences).

### Treg suppressive capacity assessment in Mixed Leukocyte Reaction Assays

Treg suppressive capacity toward proliferation of activated allogeneic carboxyfluorescein succinimidyl ester (CFSE)-labeled T lymphocytes was assessed by flow cytometry analysis after 5 days of co-culture experiments. Briefly, cord blood and healthy adult blood samples were collected after informed consent had been obtained. T lymphocytes were immunomagnetically purified from healthy donor’s peripheral blood mononuclear cells by positive selection using anti-human CD3 microbeads (Miltenyi Biotec) according to the manufacturer's instructions. These T lymphocytes were then labeled by CFDA-SE (CellTrace-CFSE cell proliferation kit, Invitrogen) by using 10 mM CFDA-SE dye to stain 10^7^ cells.

CD8^+^CD25^+^ Tregs were isolated from cord blood mononuclear cells as described above. The purity of the selected cells was always above 96%, as determined by flow cytometry analysis.

Mixed leukocyte reactions (MLR) were performed by culturing irradiated allogeneic peripheral blood mononuclear cells, as stimulating cells (2 × 10^5^), to activate CFSE-labeled allogeneic T lymphocytes responder cells (2 × 10^5^), in a 48-well plate (control MLR).

CD8^+^CD25^+^ nTregs or CD8^+^CD25^−^ T cells were added to MLRs at a 1:1:1 ratio before culture in RPMI with 10% decomplemented FBS (both from Lonza Europe, Verviers, Belgium). After 5 days of co-culture, CFSE fluorescence dilution was measured by flow cytometry, gating on CFSE positive cells. Samples were run on a FACSCalibur (BD Biosciences) and analyzed using Kaluza® Flow Cytometry Analysis software (Beckman Coulter Inc.).

### RNA extraction, RT and real-time PCR quantification

Total RNA was extracted from cells using Trizol® total RNA isolation reagent (Invitrogen, Life Technologies, Belgium). The concentration was quantified using a NanoDrop Spectrophotometer. TaqMan microRNA assays (Applied Biosystems) were used to quantify mature microRNA (miR) expression. RNU44 (Applied Biosystems) was used as endogenous control for miR expression studies.

Thus, gene-specific RT was performed for each miR using 10 ng of purified total RNA, 100 mM dNTP, 50 U MutliScribe reverse transcriptase, 20 U RNase inhibitor, and 50 nM of gene-specific RT primer samples using the TaqMan MicroRNA Reverse Transcription kit (Applied Biosystems). Fifteen microliter reactions were incubated for 30 min at 16°C, 30 min at 42°C, and 5 min at 85°C to inactivate the reverse transcriptase. Real-time RT-PCR (5 μL of RT product, 10 μL TaqMan 2× Universal PCR master Mix, (Applied Biosystems) and 1 μL TaqMan MicroRNA Assay Mix containing PCR primers and TaqMan probes) were run in triplicates at 95°C for 10 min followed by 40 cycles at 95°C for 15 s and 60°C for 1 min.

Quantitative miR expression data were acquired and analyzed using an ABI Prism 7900HT Sequence Detection System (Applied Biosystems).

### TLDA and individual quantitative PCR validation

The TaqMan Low Density Array (TLDA, Applied Biosystems) technique allowed us to measure the expression of 384 miRs, in order to compare the miR expression profile of purified nTregs with their CD25^−^ counterpart T cells originating from five different cord bloods. cDNA was synthesized from 100 ng of total cellular RNA by First Strand cDNA Synthesis System using 100 mM dNTP, 50 U MutliScribe reverse transcriptase, 20 U RNase inhibitor, 10× RT buffer, and Multiplex RT human primer. Ten microliter reactions were incubated for 30 min at 16°C, 30 min at 42°C, 5 min at 85°C to inactivate the reverse transcriptase.

Five microliter cDNA were mixed with 307 μL nuclease free water and 50 μL of this diluted cDNA were mixed with 50 μL of TaqMan Universal PCR Master Mix (Applied Biosystems). After that, 100 μL of the sample-specific PCR mixture was loaded into one sample port, the cards were centrifuged twice for 1 min at 280 *g* and sealed to prevent well-to-well contamination. The cards were placed in the Micro Fluidic Card Sample Block of an ABI Prism 7900 HT Sequence Detection System (Applied Biosystems). The thermal cycling conditions were 2 min at 50°C and 10 min at 95°C, followed by 40 cycles of 15 s at 95°C and 1 min at 60°C.

In each sample, average cycle threshold (Ct) values for the target genes were subtracted from the average Ct value of the reference gene to yield the ΔCt value. 2-ΔCt values were calculated to indicate the relative amount of transcripts in each sample.

For each miR that was found differentially expressed, a validation by individual quantitative PCR was performed, and we retained only the miR concordant with the two techniques.

### Real-time PCR

Real-time PCR quantitative mRNA analyses were performed on the ABI Prism 7000 sequence detection system using the SYBR Green fluorescence quantification system (Applied Biosystems, Warrington, UK). The standard PCR conditions were 95°C for 10 min, 40 cycles for 1 min at 94°C, 56°C (1 min), and 72°C (2 min), followed by the standard denaturation curve. The sequences of human primers were designed in Primer3 Input software (version 0.4.0) and the primers were designed as follows (Invitrogen):

β-actin,

sense: *TGACAAAACCTAACTTGCGC*,

antisense: *ATAAAGCCATGCCAATCTCA*.

IL-10,

sense: *AGATCT-CCGAGATGCCTTCA*,

antisense: *CCGTGGAGCAGGTGAAGAAT*

TGF-β,

sense: GTGGAAACCCACAACGAAA,

antisense: TAAGGCGAAAGCCCTCAAT

Foxp3,

sense: GAGAAGCTGAGTGCCATGCA,

antisense: GGTCAGTGCCATTTTCCCAG

CTLA-4,

sense: ATCGCCAGCTTTGTGTGTGA,

antisense: GACCTCAGTGGCTTTGCCTG

FOXO1,

sense: GCAGATCTACGAGTGGATGGT,

antisense: AAACTGTGATCCAGGGCTGTC

HELIOS,

sense: TCACAACTATCTCCAGAATGTCA,

antisense: AGGCGGTACATGGTGACTCAT

ICOS,

sense: CCATAGGATGTGCAGCCTTTG,

antisense: GGTCGTGCACACTGGATGAA

CD28,

sense: ATGCTCAGGCTGCTCCTGGCTCTC,

antisense: CAGCCGGCCGGCTTCTGGATAG

GARP,

sense: CCCTGTAAGATGGTGGACAAGAA,

antisense: CAGATAGATCAAGGGTCTCAGTGTCT

CCR4,

sense: GTGGTTCTGGTCCTGTTCAAATAC,

antisense: CGTGGAGTTGAGAGAGTACTTGGTT

IL2RA,

sense: AATGCAGCCAGTGGACCAA,

antisense: TGATAAATTCTCTCTGTGGCTTCATTT

The SYBR Green PCR master mix (Applied Biosystems), 0.1–0.2 μg/μl specific primers and 2.5 ng cDNA, was used in each reaction. Threshold for positivity of real-time PCR was determined based on negative controls. Calculations to determine the relative level of gene expression were made according to the instructions from the user’s bulletin (P/N 4303859) of Applied Biosystems by reference to the β-actin in each sample, using the cycle threshold (Ct) method. Negative controls without RNA and without RT were also performed. Results show one experiment representative of three.

### Cell line culture

The 293 T and HeLa cell lines were cultured in DMEM (Lonza, Verviers, Belgium) supplemented with 10% heat inactivated fetal bovine serum (Invitrogen Europe, Paisley, UK), 2 mM l-glutamine, 50 IU/mL Penicillin and 50 μg/mL Streptomycin (all from Lonza).

### Plasmid construction

A 249-bp fragment of *FOXP3* 3′-UTR encompassing the miR-335 potential target site and a 300-bp fragment of *CTLA-4* encompassing the miR-9 and miR-155 potential target sites were cloned downstream of the *Renilla* luciferase gene (Eco RI/Xho I sites) in the psiCHECK-1 plasmid (Promega, Mannheim, Germany) and designated as psiCHECK 3′-UTR WT. PCR primers used for amplification of the *FOXP3* and *CTLA-4* 3′-UTR were as follows (5′ to 3′):

*FOXP3* primers:

GCGCCTCGAGTCACCTGTGTATCTCACGCATA (forward)

GCGCGAATTCGAGCTCGGCTGCAGTTTATT (reverse)

*CTLA-4* primers:

GCGCCTCGAGAGGAGCTCAGGACACTAATA (forward)

GCGCGAATTCAATTGGGCCCATCGAACT (reverse)

QuikChange site-directed mutagenesis (deletion) of miR-9, miR-24, miR-155 and miR-335 target sites in psiCHECK 3′-UTR WT was performed according to manufacturer's protocols (Stratagene, La Jolla, CA) and designated as psiCHECK-UTRdel. QuikChange site-directed mutagenesis were performed using the following primers (5′ to 3′):

*FOXP3* (miR-335 site deleted 3′UTR):

GCCCCCCAGTGGGTGTCCCGTGCAG (forward)

CTGCACGGGACACCCACTGGGGGGC (reverse)

*CTLA-4* (miR-9 site deleted 3′UTR):

GGGAATGGCACAGCAGGAAAAGGG (forward)

CCCTGCCTTTTCCTGCTGTGCCATTCCC (reverse)

*CTLA-4* (miR-155 site deleted 3′UTR),

GGGATTAATATGGGGATGCTGATGTGGGTCAAGG (forward)

CCTTGACCCACATCAGCATCCCCATATTAATCCC (reverse)

GARP 3′UTR 2070-bp encompassing the miR-24 and −335 potential target sites were cloned downstream the *Firefly* luciferase gene (AsiSI/Xho1 sites) in the pEZX-MT01 plasmid (Labomics, Nivelles, Belgium) and designed as pEZX-MT01 3′-UTR WT. PCR primers used for amplification of the *FOXP3* and *CTLA-4* 3′-UTR were as follows (5′ to 3′):

GARP primers:

CTCGAGAGAAGCCGGGAGAC (forward)

CCGCGGACATTCAGGTAGG (reverse)

QuikChange site-directed mutagenesis were performed using the following primers (5′ to 3′):

GARP (miR-24 site deleted 3′UTR),

GCCCCACCTTGGCTGCAGGAGCTAAAACC (forward)

GGTTTTAGCTCCTGCAGCCAAGGTGGGGC (reverse)

GARP (miR-335 3′UTR site deleted),

GGGTTCTCCTGTTCTCTCTGTCATTCTCTCATTCCC (forward)

GGGAATGAGAGAATGACAGAGAGAACAGGAGAACCC (reverse)

The constructs were verified by sequencing (GATC Biotech, Konstanz, Germany).

### Luciferase assays

Luciferase assays were conducted in a 24-well format. Reporter plasmids (psiCHECK, psiCHECK 3′-UTR WT, psiCHECK 3′-UTR deleted, pEZX-MT01, pEZX-MT01 3′-UTR GARP WT, pEZX-MT01 GARP 3′-UTR deleted) (100 ng) were co-transfected in HEK293T and HeLa cells along with miR-9, miR-24, miR-155, and miR-335-mimic/miR-negative control-mimic at a final concentration of 10 μM (mirVana miRNA mimic, Life Technologies, Gent, Belgium) and control firefly plasmid pGL3-CMV for the psiCHECK vectors only (100 ng) using Lipofectamine 2000 (Invitrogen) according to the manufacturer's guidelines. Before proceeding to the transfection assays, the cell lines were assessed for expression of the miR of interest using quantitative RT-PCR, as described below. 24 h post-transfection, cells were harvested, and luciferase levels were measured using the Dual-Luciferase reporter assay system (Promega Benelux BV, Leiden, The Netherlands) according to the manufacturer's guidelines. Relative protein levels were expressed as *Renilla*/firefly luciferase ratios. Relative protein levels were expressed as *Renilla*/firefly luciferase ratios in case of co-transfections with psiCHECK and PGL3-CMV vectors and firefly/Renilla for transfections with pEZX-MT01 vectors.

### Lentiviral vector production

VSV-G pseudotyped lentiviral particles were generated by polyethyleneimine (Sigma) co-transfection of HEK293T cells with three plasmids, pMIRNA, pCMVΔR8.91, and pMD.G [60].

pCMVΔR8.91 is an HIV-derived packaging construct that encodes the HIV-1 Gag and Pol precursors as well as the regulatory proteins Tat and Rev [61]. VSV-G was expressed from pMD.G [62]. pMIRNA, provided by System Biosciences, is a lentivirus-based vector in which a microRNA precursor molecule has been cloned downstream of the CMV promoter and contains *copGFP* as a reporter gene. 24 h after transient transfection of HEK293T cells, viral supernatants were collected, filtered through 0.45-μm low protein-binding filters (Nalgene, Rochester, NY), and concentrated as described previously [63]. The viral pellets were then resuspended in 1/100 volume of PBS. Viral stocks were stored in aliquots at −80°C, and the titers were determined by transducing HeLa cells in a limiting dilution assay. Lentiviral vector preparations collected 24 h post-transfection, and after concentration, displayed titers of 10^8^–10^9^ transducing units/ml in HeLa cells. No replication-competent virus was detected in the concentrated lentiviral stocks. A second production cycle was repeated after 24 h, routinely generating lentiviral vector preparations of 5 × 10^7^ to 5 × 10^8^ transducing units/ml.

### Lentiviral transduction

Tregs were plated at a density of 10^6^ cells/well in 12-well tissue culture plates in 1 ml of RPMI 1640 supplemented with 10% heat-inactivated fetal bovine serum, 2 mM L-glutamine, 50 units/ml penicillin, 50 μg/ml streptomycin (Lonza), in the presence of 5 μg/ml phytohemagglutinin (PHA-L, Sigma) and 20 units/ml IL-2.

24 h after Treg isolation, cells were exposed to lentiviral vector preparation at a multiplicity of infection of 5 in a volume of 500 μl in the presence of 8 μg/ml polybrene (Sigma). GFP-positive cells were sorted by flow cytometry at day 7 after transduction.

### MiR-9, miR-24, miR-155 and miR-335 detection by TaqMan Real-time PCR

TaqMan miRNA assays (Applied Biosystems) used the stem loop method [64,65] to detect the expression level of mature miR-9, miR-24 miR-155 and miR-335. For RT reactions, 10 ng of total RNA was used in each reaction (15 μl) and mixed with the RT primer (3 μl). The RT reaction was carried out under the following conditions: 16°C for 30 min, 42°C for 30 min, 85°C for 5 min, and then holding at 4°C. After the RT reaction, the cDNA products were diluted at 5×, and 9 μl of the diluted cDNA was used for PCR along with the TaqMan microarray assay (1 μl) and PCR master mix (10 μl). The PCR was conducted at 95°C for 10 min, followed by 40 cycles of 95°C for 15 s and 60°C for 60 s in the ABI 7900 real-time PCR system. The real-time PCR results were analyzed and expressed as relative miRNA expression of *Ct* value, which was then converted to -fold changes. RT primer, PCR primers, and TaqMan probe for miR-9, miR-24, miR-155 and miR-335 were purchased from ABI. RNU48 was used for normalization.

### Quantitative PCR for FOXP3, CTLA-4 and GARP expression

Total RNA was extracted with Trizol reagent according to the manufacturer's guidelines (Invitrogen), and first-strand cDNAs were synthesized by reverse transcription (Superscript First-strand Synthesis System for RT-PCR kit, Invitrogen) Quantitative mRNA expression was measured by real-time PCR with the PRISM 7900 sequence detection system (Applied Biosystems), and the TaqMan master mix kit; EF1-α mRNA was used as an internal control. Human TaqMan gene expression assay for *FOXP3* (Hs01085834-m1), *CTLA-4* (Hs03044418-m1), GARP (Hs0019436-m1) and EF1-α (Hs01024875-m1) were purchased from Applied Biosystems. The program used for amplification was as follows: 10 min at 95°C followed by 40 cycles of 15 s at 95°C, 1 min at 60°C.

### Statistical analysis

Data are presented as means ± SD of at least three independent experiments and analyzed using Student's *t* test. *p* values of <0.05 (*), <0.01 (**), and <0.001 (***) were considered significant.

## Results

### CD8^+^CD25^+^ natural Treg cells express FOXP3 and CTLA-4

**Figure 1:**
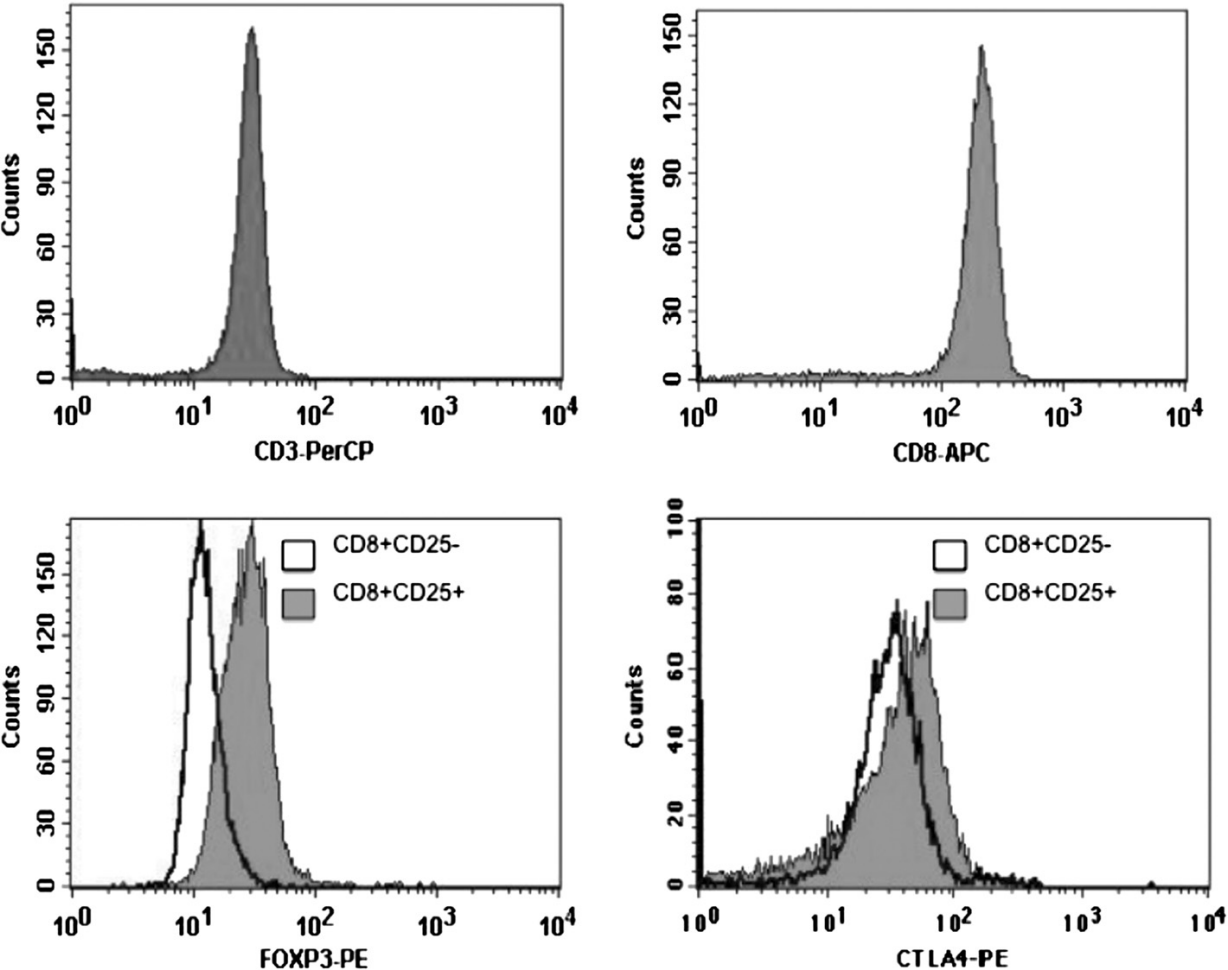
**Purified human CD8** ^**+**^ **CD25** ^**+**^ **natural Tregs express FOXP3 and CTLA-4.** Separated cell fractions, CD8^+^CD25^+^ and CD8^+^CD25^−^ T cells, were analyzed by multicolor FACS using the following antibodies: anti-CD3 PerCP, anti-CD8-APC (BD biosciences) and anti-CD25-PE (Miltenyi Biotec) to assess purity for each isolation. Intracellular FOXP3 and cell-surface CTLA-4 were assessed using human anti-FOXP3-PE detection kit and anti-CTLA-4-PE (BD biosciences). Flow cytometry was performed using a FACSCalibur applying CellQuest software (BD Biosciences). CD3 and CD8 purity was above 97% among isolated CD8^+^ T cells. CD8^+^CD25^+^ nTregs express FOXP3 and CTLA-4 when compared to CD8^+^CD25^−^ T cells.

CD8^+^CD25^+^ and CD8^+^CD25^−^ T cells were immunomagnetically purified from fresh human cord blood by negative selection of CD8^+^ T cells, followed by CD25-based positive selection (Miltenyi Biotec, Bergisch Gladbach, Germany). To confirm the purity of the separated cell fractions, CD8^+^CD25^+^ and CD8^+^CD25^−^ T cells were analyzed by flow cytometry. Purity was always >96%. CD8^+^ natural Treg cells expressed more intracellular FOXP3 and cell-surface CTLA-4 proteins compared with CD8^+^ CD25^−^ T cells (Figure 1).

### CD8^+^CD25^+^ natural Treg cells are suppressive

**Figure 2:**
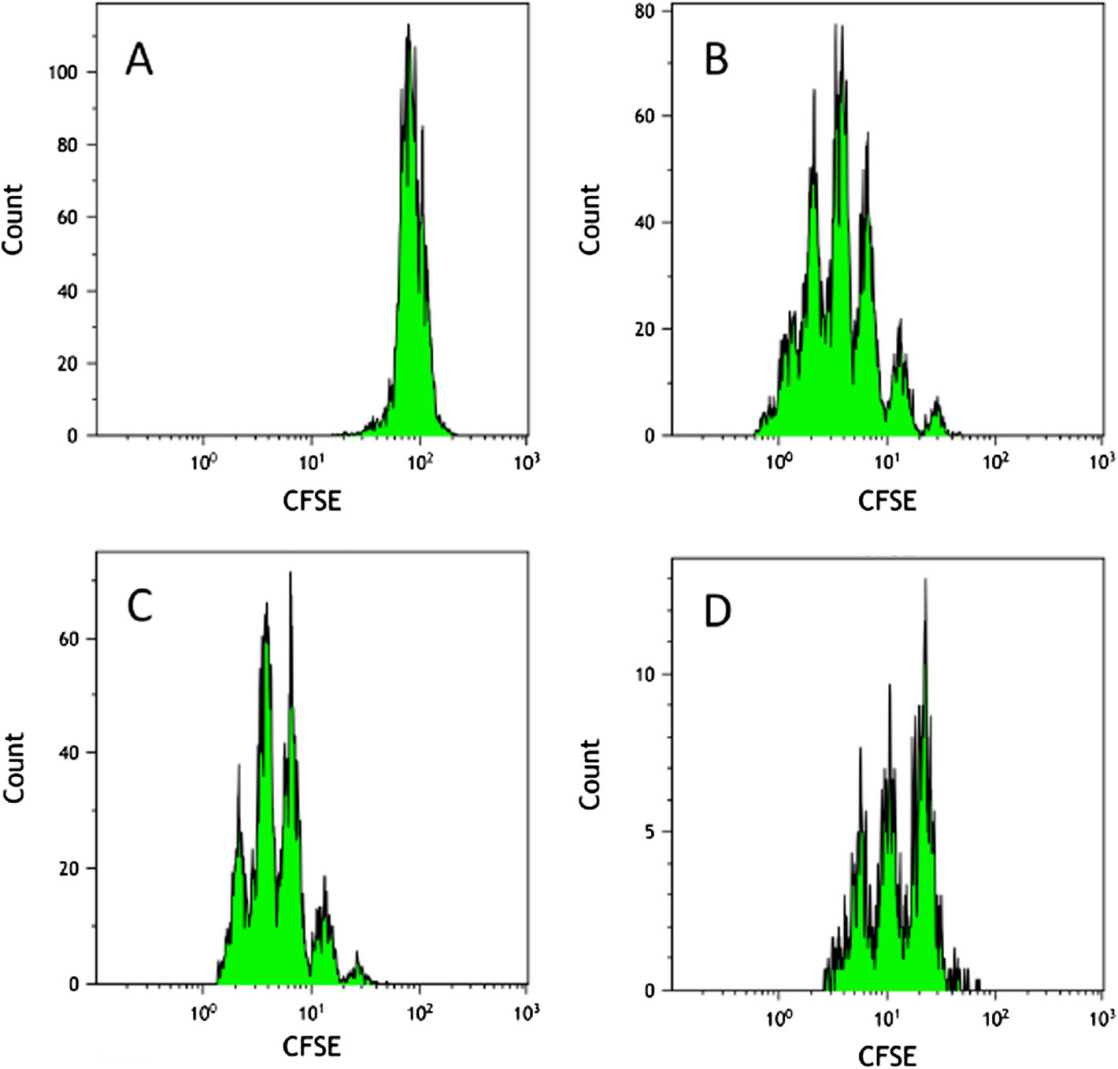
**Functional assessment of CD8** ^**+**^ **CD25** ^**+**^ **nTreg cell suppressive capacity toward proliferation of allogeneically activated CFSE-labeled T lymphocytes.** CFSE dilution analysis shows CD8^+^CD25^+^ nTreg-mediated suppression of allogeneic T cell proliferation in 5 days mixed leukocyte reaction (MLR) compared to control MLR and compared to MLR with CD8^+^CD25^−^ non-Tregs. CFSE dilution histograms are shown for 1:1 suppressor/responder cell ratio. Shown is one representative experiment out of four independent experiments performed. **(A)** CFSE-labeled allogeneic T cells alone at d0 before MLR. **(B)** Proliferation of CFSE-labeled allogeneic T cells after 5 days of allo-MLR (control MLR). **(C)** Allo-MLR in presence of CD8^+^CD25^−^ non-Treg cells, at day 5. **(D)** Allo-MLR in presence of CD8^+^CD25^+^ nTregs, at day 5.

In vitro Treg cell suppression assays were performed to assess the functional impact of the isolated Treg cells on activated T cell proliferation. CD8^+^CD25^+^ natural Treg cells suppressed allogeneic mixed leukocyte reactions when measured at day 5, as indicated by CFSE dilution of CD3^+^ T cells. Our analysis (Figure 2) indicated that CD8^+^CD25^+^ natural Treg cells could inhibit allogeneic T cell proliferation.

### Assessment of Treg cell-related gene expression in CD8^+^CD25^+^ and CD8^+^CD25^−^ T cells qPCR

The mRNA transcript levels were analyzed by qPCR to compare CD8^+^CD25^+^ with CD8^+^CD25^−^ T cells using SYBR green detection. Importantly, we showed (Table 1) that IL-2RA, FOXP3, CTLA-4, CCR4, GARP, IL-10 and TGF-β were upregulated in CD8^+^CD25^+^ natural Treg cells compared with CD8^+^CD25^−^ T cells, whereas CD28, ICOS, FOXO1 and HELIOS were downregulated in CD8^+^CD25^+^ natural Treg cells compared with CD8^+^CD25^−^ T cells.

**Table 1:** **Relative regulatory T cell-associated gene expression for CD8** ^**+**^ **CD25** ^**+**^ **/CD8** ^**+**^ **CD25** ^**−**^ **by individual qPCR (SYBR Green)**

**Relative Treg-associated gene expression**							
**Gene**	**IL2RA**	**FOXP3**	**CTLA-4**	**CCR4**	**GARP**	**IL10**	**TGF-B**
CD8 + CD25+/CD8 + CD25-	65 ± 1,414	11,5 ± 4,9	3.19 ± 1,73	13.43 ± 4,69	13,66 ± 4,12	4.86 ± 2,68	4.57 ± 2,61
**Gene**	**CD28**	**ICOS**	**FOXO1**	**HELIOS**			
CD8 + CD25+/CD8 + CD25-	0,05 ± 0,019	0,342 ± 0,125	0,18 ± 0,078	0,288 ± 0,13			

Gene mRNA levels were evaluated using quantitative RT-PCR of CD8^+^CD25^+^/CD8^+^CD25^−^ T cells. Data represent the mean ± SD of five independent experiments, each done in triplicate.

### The CD8^+^CD25^+^ natural Treg cell microRNA signature

**Figure 3:**
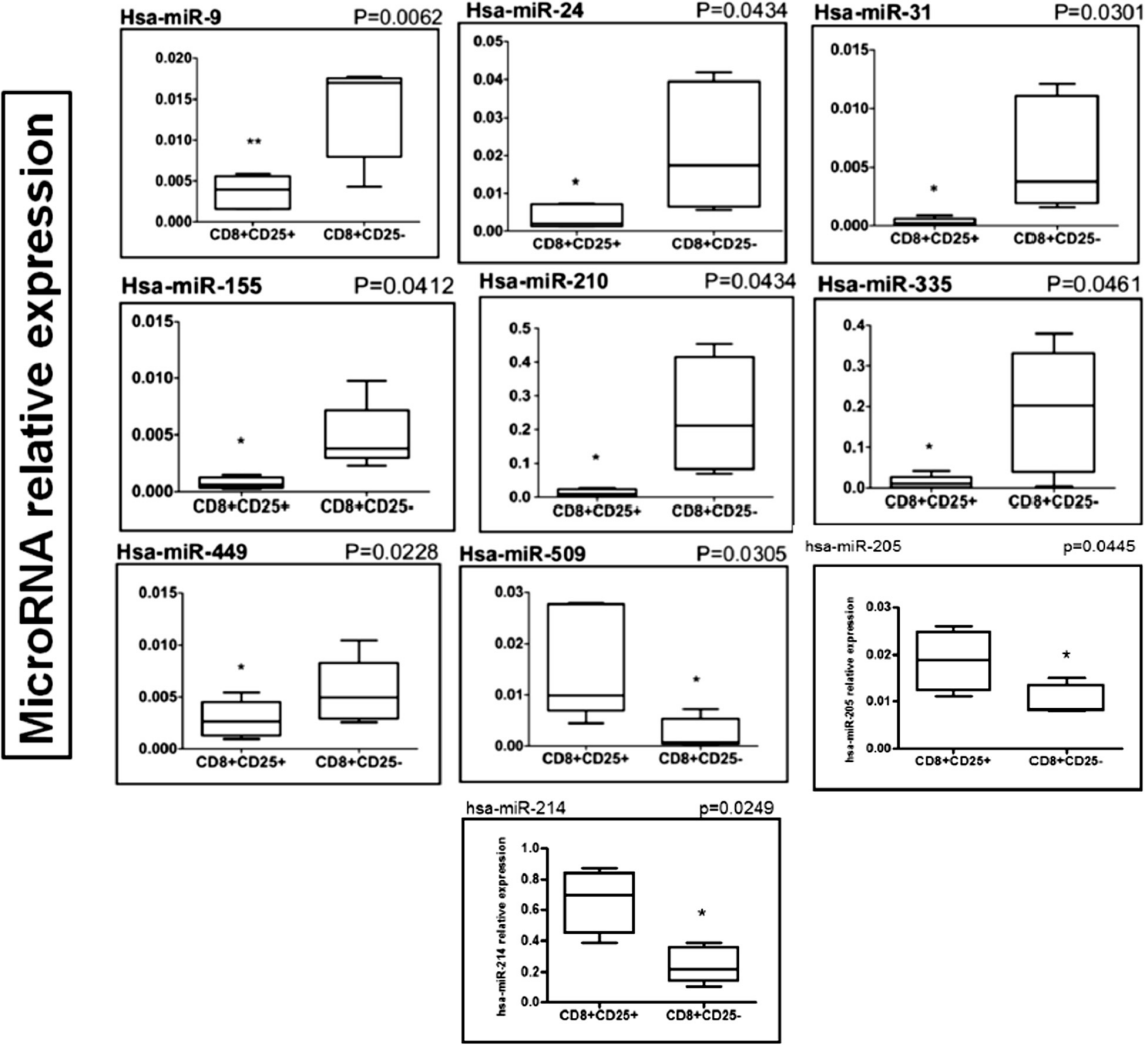
**Differential expression of miR-24, −335, −155, −31, −210, −449, −509, −214, −205 and −9 between CD8** ^**+**^ **CD25** ^**+**^ **nTregs and CD8** ^**+**^ **CD25** ^**−**^ **T cells.** Data obtained by qRT-PCR amplification of miRs are plotted. *p* values for each miRNA relative expression are presented. *Boxes* represent SE; and Error bars SD Pooled data from five independent experiments are shown. (*p < 0.05; **p < 0.01 CD8^+^CD25^+^ nTregs versus CD8^+^CD25^−^ T cells (Student's *t* test).

We first studied miRNAs from five independent CD8^+^CD25^+^ natural Treg cell and CD8^+^CD25^−^ T cell samples using the TLDA technique. We could identify several miRNAs that were differentially expressed between CD8^+^CD25^+^ natural Treg cells and CD8^+^CD25^−^ T cells. Differentially expressed miRNAs were validated by real-time PCR (Figure 3). A Treg cell miRNA signature was identified that included 10 significantly differentially expressed miRNAs: miR-9, −24, −31, −155, −210, −335 and −449 were downregulated in CD8^+^ natural Treg cells, whereas miR-214, −205 and −509 were overexpressed.

### Target prediction for miR-9, −24, −155 and −335

Computer-based programs were used to predict potential targets sites for the miRNAs that were associated with downregulation in the *FOXP3* and *CTLA-4* 3′-UTRs. We searched miRBase [66] and TargetScan 4.2 [67]. We could identify putative miRNA target sites in the *FOXP3* and *CTLA-4* 3′-UTRs by both programs—miR-335 in the *FOXP3* 3′-UTR and both miR-9 and −155 in the *CTLA-4* 3′-UTR. Furthermore, miR-31, −24, −210 and −335 have target sites in the *FOXP3* 3′-UTR but not in the *CTLA-4* 3′-UTR, while miR-9 and −155 have target sites in the *CTLA-4* 3′-UTR but not in the *FOXP3* 3′-UTR [68,69].

### FOXP3 is directly regulated by miR-335

**Figure 4:**
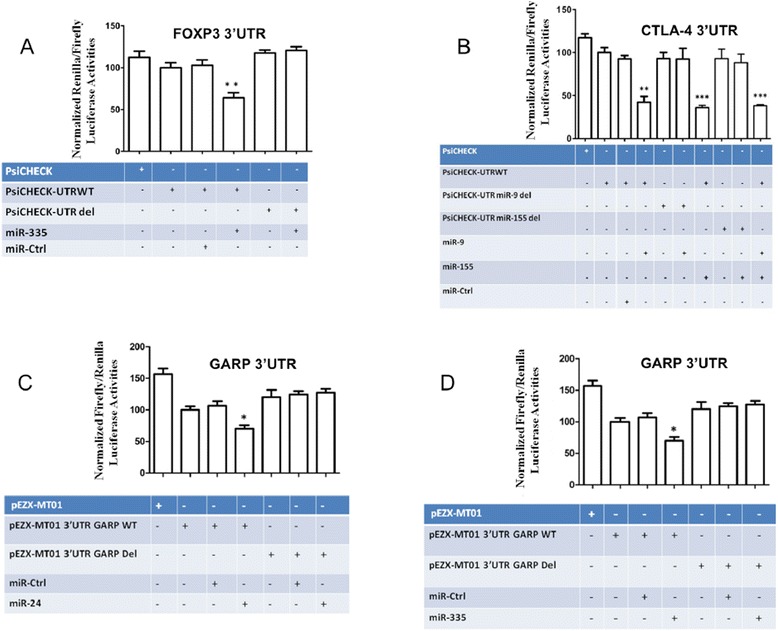
**MicroRNA specific activities. (A)** MiR-335 negatively regulates luciferase expression in a plasmid coupling its coding sequence with FOXP3 3′UTR. *Renilla* luciferase reporter assays with constructs holding *FOXP3* 3′-UTR sequences from the indicated genes were co-transfected into HEK293T cells along with a firefly luciferase transfection control plasmid either alone or together with miR-335. **(B)** MiR-9 and miR-155 negatively regulate luciferase expression in a plasmid coupling its coding sequence with CTLA-4 3′UTR. *Renilla* luciferase reporter assays with constructs holding *CTLA-4* 3′-UTR sequences, wild type or miR-site deleted, were co-transfected into HEK293T cells along with a firefly luciferase transfection control plasmid either alone or together with miR-9, −155. MiR-24 **(C)** and miR-335 **(D)** specifically targets GARP 3′UTR and negatively regulate luciferase reporter expression. *Renilla* and *firefly* luciferase reporter assays with constructs holding *GARP* 3′-UTR sequences, wild type or miR-site deleted, were co-transfected into HEK293T cells along with miR-24, −335. Relative luciferase values normalized to transfections without miRNA are shown. Data represent mean ± SD (*error bars*) of three independent experiments, each performed in triplicate. (*p < 0.05; **, p < 0.01, Student's *t* test).

A 249-bp fragment of the 3′-UTR of *FOXP3* containing the miR-335 target sequence was cloned into a psiCHECK-1 vector downstream of the *Renilla* luciferase gene (psiCHECK-UTRwt). In parallel, in the same way we cloned this *FOXP3* 3′-UTR fragment with a deleted miR-335 target site (psiCHECK-UTRdel). Transient transfections of psiCHECK-UTRwt or psiCHECK-UTRdel in HEK293T cells led to no significant change in reporter luciferase activity when compared with the psiCHECK control vector (Figure 4A). By contrast, co-transfection of HEK293T cells with miR-335 and psiCHECK-UTRwt led to a significant reduction (~47%) in relative reporter luciferase activity, and also for activity compared with the co-transfection of HEK293 T cells with miR-Ctrl and psiCHECK-UTRwt.

Together, these results demonstrate a specific role for miR-335 in the regulation of *FOXP3* expression through direct binding to its target site.

### CTLA-4 is directly regulated by miR-9 and miR-155

We next investigated whether *CTLA-4* could be directly targeted by miR-9 and −155. We engineered luciferase reporter plasmids containing either the wild-type 3′-UTR of this gene (psiCHECK-UTRwt) or a 3′-UTR with deleted miR-9 and −155 target sites (psiCHECK-UTRdel). We found that co-transfection of HEK293 T cells with miR-9 and psiCHECK-UTRwt led to a reduction (~52%) in reporter luciferase activity compared with the co-transfection of HEK293 T cells with miR-9 and psiCHECK-UTRdel or the co-transfection of HEK293 T cells with miR-Ctrl and either psiCHECK-UTRwt or psiCHECK-UTRdel (Figure 4B).

Similarly, the co-transfection of HEK293 T cells with miR-155 and psiCHECK-UTRwt led to a significant reduction (~65%) in reporter luciferase activity, while there was no difference observed for the co-transfection of HEK293 T cells with miR-155 and psiCHECK-UTRdel or for the co-transfection of miR-Ctrl and either psiCHECK-UTRwt or psiCHECK-UTRdel. Moreover, the co-transfection of HEK293 T cells with psiCHECK-UTRwt and both miR-9 and −155 led to a reduction (~65%) in reporter luciferase activity, but this reduction was not greater than that achieved using miR-155 alone, suggesting that either miR-155 or −9 alone could achieve robust downregulation of CTLA-4.

Altogether, these results demonstrate the roles of miR-9 and −155 in the regulation of CTLA-4 expression through direct and specific binding to their target sites.

### The *GARP* 3′-UTR is directly targeted by miR-24 and −335

Analysis of reporter luciferase activity in HEK293 T cells co-transfected with the *GARP* 3′-UTR, wild-type or miRNA site-deleted, and either miR-24 (Figure 4C) or miR-335 (Figure 4D), showed a direct and specific targeting of the *GARP* 3′-UTR by these miRNAs, leading to reduced luciferase expression.

### Lentiviral expression of miR-335 in fresh Treg cells significantly reduces *FOXP3* transcription

**Figure 5:**
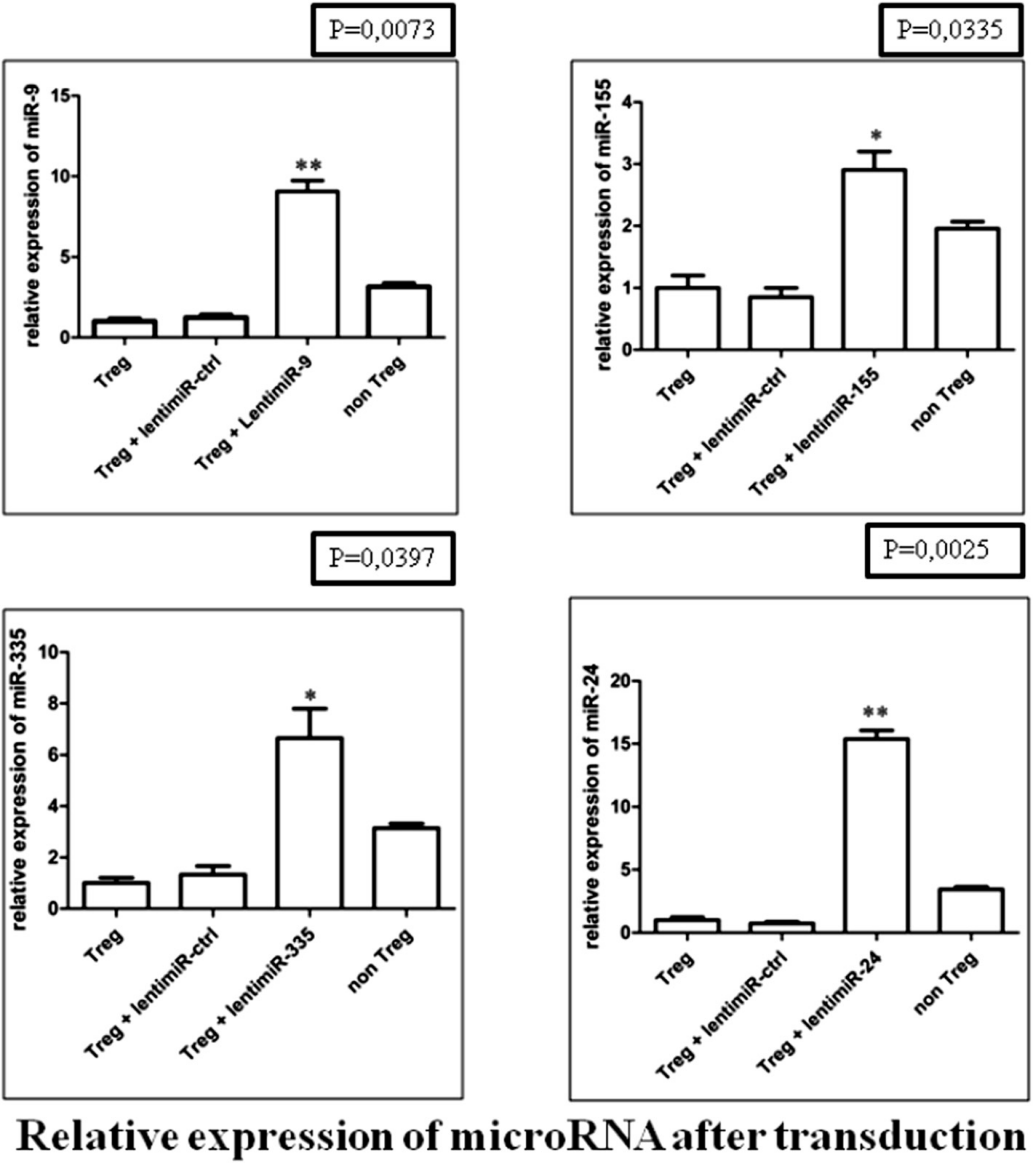
**Differential expression of miR-24, −335, −155 and −9 in CD8** ^**+**^ **CD25** ^**+**^ **natural Treg cells after transduction by lenti-miR-24, −335, −155 and −9.** Data obtained by qRT-PCR amplification of miRs are plotted for CD8^+^CD25^+^nTregs versus non-transduced CD8^+^CD25^+^ nTregs. *p* values for each miRNA relative expression are presented. *Boxes* represent SE; and Error bars SD. Pooled data from five independent experiments are shown. (*p < 0.05; **p < 0.01 Student's *t* test).

**Figure 6:**
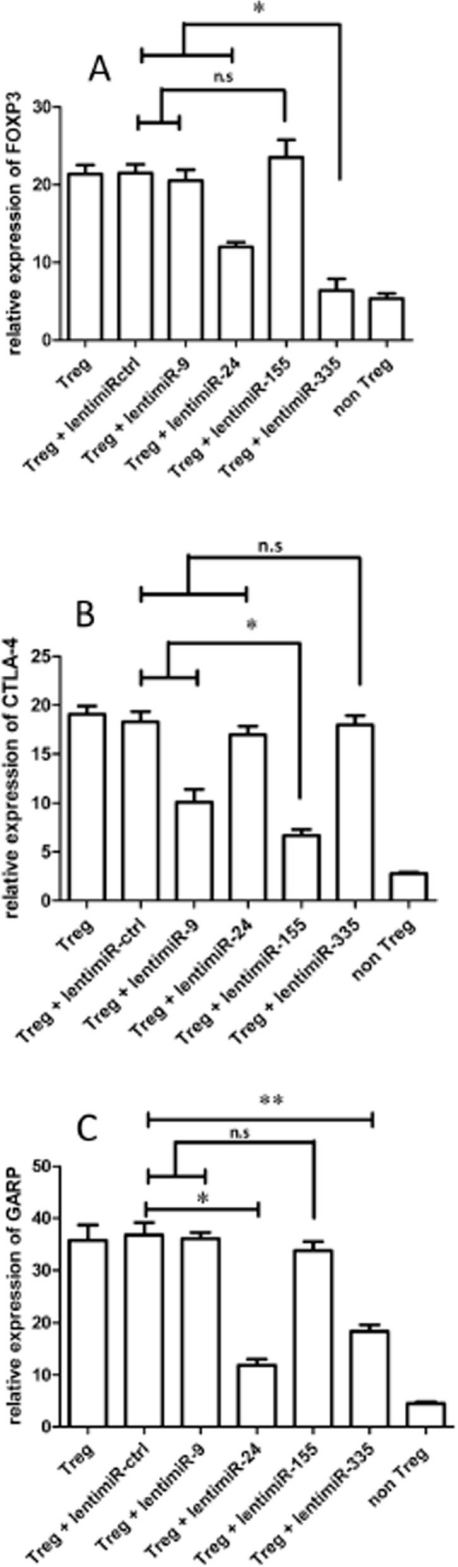
**MicroRNA specific activities. (A)** In primary human Treg cells, miR-335 specifically regulates FOXP3 expression. Relative miR-335 and FOXP3 expression in CD8^+^CD25^+^ Treg cells transduced by lenti-miR-335 compared with CD8^+^CD25^+^ Treg cells transduced by lenti-miR-Ctrl, as determined by relative qRT-PCR. **(B)** In primary human Treg cells, miR-9 and −155 specifically regulate CTLA-4 expression. Relative miR-9, miR-155 and CTLA-4 expression in CD8^+^CD25^+^ Treg cells transduced by lenti-miR-9 or lenti-miR-155 compared with CD8^+^CD25^+^ Treg cells transduced by lenti-miR-Ctrl, as determined by relative qRT-PCR. **(C)** In primary human Treg cells, miR-24 and −335 specifically regulate GARP expression. Relative miR-24 and GARP expression in CD8^+^CD25^+^ Treg cells transduced by lenti-miR-24 compared with CD8^+^CD25^+^ Treg cells transduced by lenti-miR-Ctrl, as determined by relative qRT-PCR. Relative miR-335 and GARP expression in CD8^+^CD25^+^ Treg cells transduced by lenti-miR-335 compared with CD8^+^CD25^+^ Treg cells transduced by lenti-miR-Ctrl. as determined by relative qRT-PCR. CD8^+^CD25^−^ T cells were considered to be non-Tregs; *p < 0.05, **p < 0.01, ***p < 0.001 (as determined by Student's *t*-test).

To study the effect of over-expression of miR-335 in Treg cells, specifically on FOXP3 expression, lenti-miR-335 was used to transduce Treg cells (Figure 5); transduced Treg cells were FACS-sorted based on GFP positivity. The miR-335 levels in lenti-miR-335-transduced Treg cells were significantly higher than in lenti-miR-ctrl transduced cells, whereas the levels of miR-335 were two-fold lower in Treg cells compared with their negative counterparts (Figure 6A). The control for each miRNA transduction was a scrambled miR sequence. Clearly, the expression levels of miR-335 in transduced Treg cells were higher than the physiological level observed in non-Treg cells, but this resulted from our inability to adjust the levels of expression of the transgene. Nevertheless, this finding indicates that miR-335 can down-regulate FOXP3 expression, although not to the level of non-Treg cells, demonstrating that FOXP3 expression must be controlled by combined inputs from several pathways, including miR-335. Additionally, the levels of FOXP3 were 3.19-fold lower in lenti-miR-31 transduced cells compared with lenti-miR-ctrl and non-transduced cells [68]. These results demonstrate that FOXP3 expression is controlled by miR-335.

### Lentiviral transduction of miR-9 and −155 in Treg cells significantly reduces CTLA-4 expression

To study the effect of miR-9 and −155 on the mRNA expression levels of *CTLA-4*, we increased miR-9 and −155 levels by expressing the miR-9 and −155 precursors from a lentiviral vector (lenti-miR-9 and −155). After lenti-miR-9 and −155 viral transduction (Figure 5), we found that the mRNA levels of *CTLA-4* decreased (2.02-fold) in the lenti-miR-9-transduced Treg cells compared with the lenti-miR-ctrl transduced cells and the mRNA levels of *CTLA-4* decreased (2.79-fold) in the lenti-miR-155 transduced Treg cells compared with the lenti-miR-ctrl transduced cells (Figure 6B).

### Lentiviral transduction of miR-24 and −335 in Treg cells significantly reduces GARP expression

Efficient ex vivo transduction of Treg cells using lenti-miR-24 and lenti-miR-335 (Figure 5) showed that miR-24 and −335 expression significantly reduced GARP expression levels by 3.21- and 1.96-fold, respectively (Figure 6C).

## Discussion

We previously described a miRNA signature in human natural CD4^+^ Treg cells. We could also identify a miRNA signature in CD4^+^CD25^+^CD127^low^ Treg cells from peripheral blood. Importantly, for both signatures, we could show how the described miRNAs specifically regulate genes associated with Treg cell function in human primary T cells [68,69].

Here, we purified CD8^+^CD25^+^ natural Treg cells from human cord blood, assessed FOXP3 and CTLA-4 expression by flow cytometry and measured mRNA levels using quantitative PCR. As expected, FOXP3 and CTLA-4 protein and mRNA levels were increased in the CD8^+^CD25^+^ natural Treg cells compared with CD8^+^CD25^−^ T cells. Importantly, those cells showed suppressive properties ex vivo in a mixed T cell reaction in which irradiated allogeneic PBMCs were used as stimulators.

Reviewing the Treg cell literature, we identified a list of genes known to play an important role in the regulation of Treg cell function and development. We investigated the relative expression of these genes in CD8^+^CD25^+^ natural Treg cells compared with CD8^+^CD25^−^ T cells using qPCR. We found that expression of the *FOXP3*, *CTLA-4*, *CCR4*, *GARP*, *IL-10* and *TGF-β* genes was upregulated in CD8^+^CD25^+^ natural Treg cells, whereas the *CD28*, *ICOS*, *FOXO1* and *HELIOS* genes were underexpressed. Obviously, *IL2RA* (CD25) was found to be overexpressed by these Treg cells. Overall, these results further support the regulatory features and potential of these cells.

Using TaqMan low-density arrays and quantitative PCR confirmation of selected transcripts, we could define the first miRNA ‘signature’ for human purified CD8^+^CD25^+^ natural Treg cells. This ‘signature’ included 10 significantly differentially expressed miRs: miR-214, −205 and −509 were overexpressed, whereas miR-9, −24, −31, −155, −335, −210 and −449 were underexpressed in CD8^+^CD25^+^ natural Treg cells compared with CD8^+^CD25^−^ T cells.

Next, we investigated the potential role of the miRNAs from this signature on the expression of genes related to Treg cell function and development. We found that the 3′-UTR of FOXP3 contained miRNA target sites for miR-24, −31, −210 and −335, which were all underexpressed in CD8^+^CD25^+^ natural Treg cells compared with CD8^+^CD25- T cells. Scanning the 3′-UTR of *CTLA-4* revealed that miR-9 and −155 (both underexpressed in Treg cells) contained a target site. Similarly, we found miR-24 and miR-335 target sites in the *GARP* 3′-UTR.

Although not Treg cell-specific, FOXP3 remains the best-known transcription factor that specifically orchestrates a transcriptional program that is required for the establishment, maintenance and function of the Treg cell lineage.

Importantly, we have previously shown that miR-24, miR-210 and miR-31 [68,69] negatively regulate the expression of FOXP3 in human T cells. Interestingly, using 3′-UTR cloning, site-directed mutagenesis and miRNA co-transfection experiments, we demonstrated that miR-335 negatively regulates FOXP3 expression in a direct and specific manner. Moreover, transducing lenti-miR-335 in natural Treg cells leads to the negative regulation of FOXP3 expression compared with lenti-miR-Ctrl transduced cells. Importantly, four out of the seven underexpressed miRNAs in the CD8^+^ natural Treg cell miRNA ‘signature’ can regulate FOXP3 expression in primary Treg cells.

Many studies have focused on the role of CTLA-4 expression in Treg cells. Kolar et al. showed that CTLA-4 had an important function in Treg cell homeostasis and also highlighted the importance of CTLA-4 in the in vivo suppressive mechanism employed by Treg cells. These findings have identified CTLA-4 as a crucial costimulator of Treg cells by demonstrating its role in mediating the resistance of Treg cells to activation-induced cell death [70]. Additionally, CTLA-4 expression and signaling are essential for Treg cells to execute their suppressive function in vivo. Moreover, CTLA-4 has been described to play a negative role in tumor progression or persistence. These findings have brought several groups, including Jim Allison’s group, to work on anti-CTLA-4 antibodies for cancer immunotherapy that have already shown very promising clinical results [70]. Considering the important role of CTLA-4 in Treg cell biology, we decided to investigate the effect of miR-9 and −155 (underexpressed in CD8^+^ natural Treg cells) on CTLA-4 expression. Using the same strategy as we did for FOXP3, we carried out *CTLA-4* 3′-UTR cloning, site-directed mutagenesis and miRNA co-transfection experiments with a reporter luciferase activity assay and lentiviral-mediated microRNA transduction of primary natural Treg cells. We could show that both miR-9 and −155 negatively regulate CTLA-4 expression in a direct and specific way. Recently, miR-155 was shown to be overexpressed in tissues of patients with atopic dermatitis, associated with inflammatory CD4^+^ T cells and capable of targeting *CTLA-4*. This study supports our findings by suggesting that miR-155 underexpression in CD8^+^ natural Treg cells contributes to the regulation of CTLA-4 expression.

GARP received significant attention from the immune regulation field, as it is naturally produced by Treg cells and has the capacity to present latent TGF-β on the Treg cell surface. Very recently, Skapenko’s group showed that miR-142-3p targeted GARP in CD4^+^CD25^+^ T cells [55] and Sophie Lucas’ group described that GARP is regulated by microRNAs (miR-142-3p, −181a and −185) and controls latent TGF-β production by human CD4^+^ Treg cells [71]. Our experiments show that miR-24 and −335 specifically bind to the *GARP* 3′-UTR and directly regulate GARP expression in primary human CD8^+^ natural Treg cells.

With great interest, we noticed the relatively high number of potential target sites for miRNAs in the signature that were found in many genes relevant to Treg cell biology. However, additional work will be required to prove that these miRNAs effectively regulate these genes.

In parallel, it is also interesting to note that the majority of miRNAs in the CD8^+^CD25^+^ natural Treg cell signature are also found to be differentially expressed in either the CD4^+^CD25^+^ natural Treg cell (miR-31) or the circulating peripheral blood CD4^+^CD25^+^CD127^low^ Treg cell (miR-9, −24, −210, −335 and −509) signatures, which may suggest that a limited number of miRNAs control the expression of major features of Treg cells. While subpopulations of Treg cells that our group has studied do not encompass all human Treg cells described, they likely cover the vast majority of them.

Conversely, the miRNAs that are not shared by several Treg cell subpopulations could condition, or be conditioned by, phenotypic or functional differences between types of regulatory cells. In this sense, miR-155 appears in our miRNA signature to be more closely linked to CD8^+^ natural Treg cells. It appears that miR-155 has an important role in the immune system. Romero’s group described a specific important role for miR-155, in mice and humans, in the maturation of CD8^+^ T cells from a naive to effector state and in their further acquisition of cytolytic properties; both processes correlated with high expression of miR-155 [72]. Our observation that miR-155 targets CTLA-4 in CD8^+^ T cells can extend the insights provided by this group, suggesting an additional role for miR-155 that not only coordinates the expression of the machinery elements needed for cytotoxicity, but also controls some immunosuppressive molecules during the effector phase of the cellular immune response.

Another observation shows that in a single subtype of human Treg cells, different microRNAs can target the 3′-UTR of the same gene (*FoxP3* is targeted by miR-24, −31, −210 and −335, *CTLA-4* is targeted by miR-9 and −155, and *GARP* is targeted by miR-24 and −335). Furthermore, a defined miRNA can target several genes important for Treg cells, suggesting a high level of coordination between miRNAs for the efficient regulation of a target gene or function. Our findings also suggest the existence of a ‘program’ carried out by a single miRNA that targets a set of genes associated with a type of activity.

All of these observations emphasize the importance of miRNA-mediated regulation of complex biological processes.

Caution should be taken when comparing miRNA signatures because they are always generated by relative comparisons to a cell type that is chosen and described as the negative counterpart of the cell population studied. This implies that a signature may vary when one swaps the cell taken as a ‘counterpart’. Therefore, it is crucial not to limit oneself to the study of how miRNAs affect the expression of a reporter gene transfected in a cell line, but rather to look at the biological relevance of the signature in the corresponding fresh primary Treg cells using transduction by a vector expressing the miRNA of interest compared with a control vector.

## Conclusions

We have identified an easy and convenient source of human CD8^+^CD25^+^ natural Treg cells and confirmed they share key known features of Treg cells. We reported for the first time the microRNA signature of these cells. Moreover, we found an essential role for six miRNAs out of 10 found in the signature, suggesting that this signature is directly relevant for Treg biology. The hypothesis that different signatures modulate distinct aspects of regulatory function should be explored. We are currently studying the role of these under- and overexpressed miRNAs to identify new target genes implicated in the direct or indirect control of Treg cell biology. While much work still needs to be done to better understand the overall regulation of Treg cell functions, this study underlines the importance of miRNAs in this area and the chance that they offer to explore new therapeutic targets in immune disorders, infectious diseases and cancers.
